# THE ASSOCIATIONS BETWEEN LONELINESS, SOCIAL ISOLATION AND FIVE DOMAINS OF COGNITION

**DOI:** 10.1093/geroni/igae098.3736

**Published:** 2024-12-31

**Authors:** Dokyung Yoon, Elizabeth Zelinski, Teal Eich

**Affiliations:** University of Southern California, Leonard Davis School of Gerontology, Los Angeles, California, United States; University of Southern California, Los Angeles, California, United States; USC, Los Angeles, California, United States

## Abstract

This study aimed to investigate the associations between loneliness, social isolation, and five cognitive domains by using a comprehensive neuropsychological battery. Participants included 2,884 older adults (mean age = 72.8) in the 2016 Health and Retirement Study Harmonized Cognitive Assessment Protocol. The 11-item UCLA Loneliness Scale was used for loneliness. For social isolation, several indicators were scaled (e.g., marital status, contact frequency with children, other family members, and friends, and participation in social organizations) using the Steptoe’s Social Isolation Index. Based on the confirmatory factor analysis findings from Zahodne et al. (2020)’s article, the average z-scores of the test results corresponding to each cognitive domain (memory, executive function, visuoconstruction, language, and processing speed) were calculated. Multiple regression for each cognitive domain was conducted. The results revealed that there were significant associations between loneliness and the cognitive scores across all five domains, indicating that the lonelier older adults were, the lower their cognitive scores tend to be. Even after controlling for demographics and depression, these associations remained significant for memory, executive function, and language. Social isolation showed a significant association only with processing speed. However, the interaction term between loneliness and social isolation was not significant across all five cognitive domains. This study provides that links between social isolation, loneliness, and cognition differ across domains. Furthermore, loneliness might have an association with memory, executive function, and language independently of depression.

